# Machine learning algorithm for early-stage prediction of severe morbidity in COVID-19 pneumonia patients based on bio-signals

**DOI:** 10.1186/s12890-023-02421-8

**Published:** 2023-04-14

**Authors:** Seung Min Baik, Kyung Tae Kim, Haneol Lee, Jung Hwa Lee

**Affiliations:** 1 Department of Critical Care Medicine, Ewha Womans University Mokdong Hospital, Ewha Womans University College of Medicine, Seoul, Republic of Korea; 2 Department of Surgery, Ewha Womans University Mokdong Hospital, Ewha Womans University College of Medicine, Seoul, Republic of Korea; 3 Department of Surgery, Korea University College of Medicine, Seoul, Republic of Korea; 4 The ECG, Inc, Seoul, Republic of Korea; 5 Department of Artificial Intelligence, Ulsan National Institute of Science and Technology, Ulsan, Republic of Korea; 6 Department of Neurology, Ewha Womans University Mokdong Hospital, Ewha Womans University College of Medicine, Seoul, Republic of Korea

**Keywords:** COVID-19, Machine learning, Bio-signal, Morbidity, Prediction

## Abstract

**Background:**

Paralysis of medical systems has emerged as a major problem not only in Korea but also globally because of the COVID-19 pandemic. Therefore, early identification and treatment of COVID-19 are crucial. This study aims to develop a machine-learning algorithm based on bio-signals that predicts the infection three days in advance before it progresses from mild to severe, which may necessitate high-flow oxygen therapy or mechanical ventilation.

**Methods:**

The study included 2758 hospitalized patients with mild severity COVID-19 between July 2020 and October 2021. Bio-signals, clinical information, and laboratory findings were retrospectively collected from the electronic medical records of patients. Machine learning methods included random forest, random forest ranger, gradient boosting machine, and support vector machine (SVM).

**Results:**

SVM showed the best performance in terms of accuracy, kappa, sensitivity, detection rate, balanced accuracy, and run-time; the area under the receiver operating characteristic curve was also quite high at 0.96. Body temperature and SpO_2_ three and four days before discharge or exacerbation were ranked high among SVM features.

**Conclusions:**

The proposed algorithm can predict the exacerbation of severity three days in advance in patients with mild COVID-19. This prediction can help effectively manage the reallocation of appropriate medical resources in clinical settings. Therefore, this algorithm can facilitate adequate oxygen therapy and mechanical ventilator preparation, thereby improving patient prognosis, increasing the efficiency of medical systems, and mitigating the damage caused by a global pandemic.

## Background

In December 2019, pneumonia of an unknown cause was detected in Wuhan, China. In February 2020, the World Health Organization officially confirmed that it was a severe acute respiratory syndrome caused by the 2019 coronavirus (COVID-19). Since then, the COVID-19 outbreak is still ongoing. Despite the development and distribution of vaccines, the prevalence of COVID-19 has not decreased owing to mutations. As of December 2021, the number of COVID-19 confirmed cases worldwide has exceeded 288 million with approximately 5 million deaths, and in South Korea, it has exceeded 630,000 cases with approximately 6,000 deaths [1]. Despite many efforts globally, the number of critically ill patients is increasing. Approximately, 15%–29% of COVID-19 cases require hospitalization, and approximately 17%–35% of hospitalized patients require intensive care [2–5]. In fact, the shortage of hospital beds and intensive care units (ICU) has emerged as a major issue worldwide, including in South Korea [6, 7]. Owing to the highly contagious nature of COVID-19, the treatment required needs to be carried out in a negative-pressure room with medical staff in a mandatory highest level of protective equipment. This has resulted in a significant burden on the healthcare system, worsened by an increased risk of infection to non-COVID-19 patients.

Currently, machine learning (ML) research on diagnosis of COVID-19 and mortality prediction is being actively conducted. A study has reported that ML methods can be applied to predict acute respiratory distress syndrome in patients with COVID-19 [8]. In addition, studies that apply ML to predict mortality and severity of COVID-19 have been steadily published in the past two years [9–11].

If the prognosis is predicted simultaneously with the COVID-19 diagnosis, limited medical resources can be allocated more efficiently. This can ultimately result in reduced mortality rates. This study aimed to apply ML to predict severe morbidity using the initial clinical information and laboratory results of COVID-19 patients. Thus, this study intends to help patients with a high probability of becoming critically ill at an early stage through timely intervention and thereby achieving efficient utilization of medical resources such as hospital beds.

## Methods

### Patient and data collection

This study was conducted retrospectively at a single institution in Korea. We included patients with mild COVID-19 requiring hospitalization from July 2020 to October 2021 and divided them into mild and exacerbation group. Data were collected retrospectively from electronic medical records (EMR) of patients. The collected clinical information and laboratory results of the patients were as follows: sex, age, height, body weight, comorbidity, duration of symptoms before hospitalization, systolic blood pressure (SBP), diastolic blood pressure, pulse rate (PR), respiratory rate, body temperature (BT), percutaneous oxygen saturation (SpO2), white blood cell count, C-reactive protein, blood urea nitrogen (BUN), total bilirubin, and procalcitonin. Cases in which the above data were omitted were excluded from this study. The criterion for exacerbation to a tertiary hospital was hypoxia that could not be maintained with O2 supply by a low-flow system, which was determined as worsening. All numerical data were collected before discharge or exacerbation, and cases collected over seven consecutive days were included in the study. The additionally created derived variables are as follows: 1) Index of whether the maximum BT three days before discharge increased compared to that of four days prior; 2) Index of whether the minimum SpO2 three days prior to discharge increased compared to four days prior; 3) ‘Vital bad index’ representing the sum of bad BT scores, bad PR scores, and bad SpO2 scores. The definitions for each score are as follows. Vital bad index: sum of bad BT score, bad PR score, and bad SpO2 score. Bad BT score is defined as 1 if the body temperature exceeds 38.5° three days before exacerbation. Bad PR score is defined as 1 if the heart rate was less than 60 bpm or greater than 110 bpm three days before exacerbation. Bad SpO2 score is defined as 1 if SpO2 was less than 93% three days before exacerbation.

### Data pre-processing

Data from patients with a hospital stay of seven days or less were excluded. Missing values were removed through data cleaning. Data matrix was created by data integration, data transformation, data reduction, and data discretization. Patients were randomly assigned to a training and a test set at a ratio of 7:3.

### Machine learning analysis

The analysis methods used in ML are random forest (RF), RF (ranger), gradient boosting machine (GBM), and support vector machine (SVM). Accuracy, kappa, specificity, precision, detection rate, balanced accuracy, and run time were calculated and compared using this model. The ability of each model to predict exacerbation was assessed by calculating the area under the receiver operating characteristics (AUROC). We divided the enrolled patients into a 70% training set and a 30% testing set. In addition, K-fold cross-validation (n_split:10) was performed to prevent data loss during model development training and to improve model prediction performance.

We quantified the uncertainty of our classification models and improved the confidence of our study by adding conformal prediction-based techniques such as class-conditional inductive conformal classifier for multi-class problems [12–14]. We split the dataset into 30% testing, 63% training, and 7% validation sets. Then we can get 99.5%, 99.7%, 99.5% accuracy of the region predictor, 0.98, 0.91, 0.97 oneC score and 1.01, 1.08, 1.03 avgC score in 95% confidence range in the order of GBM, RF and SVM.

### Statistical analysis

Numeric and categorical variables were compared using t-tests and Chi-squared tests, as appropriate. Statistical analyses were performed using RStudio (version 4. 1. 2; Boston, MA, USA).

## Results

### Baseline characteristics

**Fig. 1:**
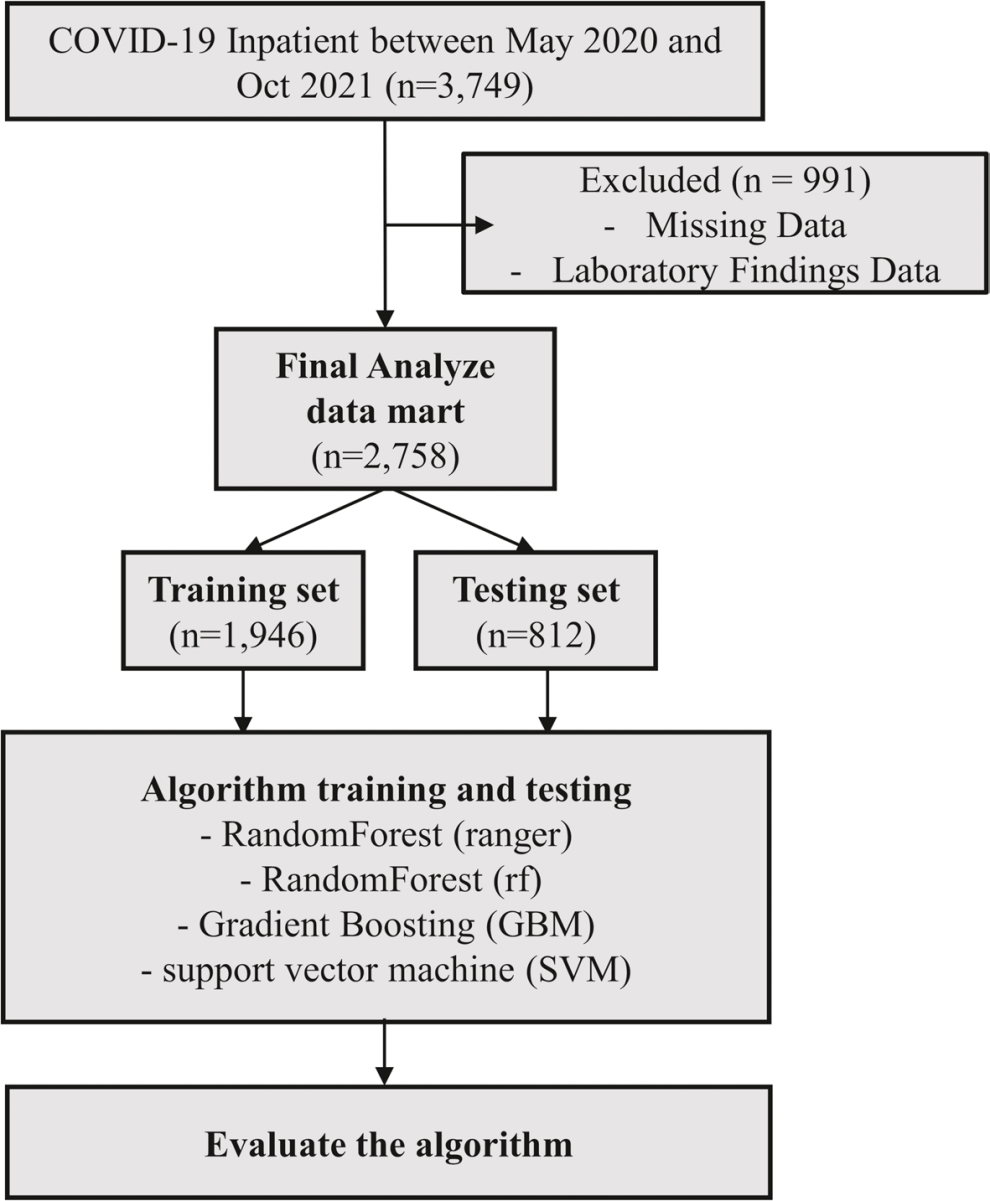
Flowchart of patient selection and model development

Of the 3744 patients diagnosed with COVID-19 by polymerase chain reaction test, 2,758 patients were finally enrolled. A total of 991 patients whose hospitalization was less than 7 days and for whom EMR data and laboratory findings were missing were excluded. Finally, the enrolled patients were randomly allocated to the training set (*n* = 1946, 70.6%) and test set (*n* = 812, 29.4%) (Fig. 1). Mild and exacerbation groups were 2696 (97.8%) and 62 (2.2%), respectively. The clinical characteristics and laboratory findings of both groups at the time of admission are shown in Table 1. In the mild and exacerbation groups, 1401 (52%) and 35 (57%) patients were male, respectively, (*P* = 0.061). The mean age of the patients in the two groups was 48.4 and 55.0 years (*P* < 0.001), respectively. The presence of co-morbidity was higher in the exacerbation group (75.8% vs. 47.2%, *P* < 0.001). SpO_2_ was 96.2% in the exacerbation group and 97.0% in the mild group (*P* = 0.017). SBP was 136.8 mmHg in the mild group, which was higher than 129 mmHg in the exacerbation group (*P* < 0.001). Among laboratory findings, the BUN level was higher in the exacerbation group than that in the mild group (14.2 mg/dL:11.9 mg/dL, respectively, *P* < 0.001).

**Table 1:** Baseline characteristics between transfer and non-transfer group

Variables	Total (*n* = 2758)	Exacerbation group (*n* = 62)	Mild group (*n* = 2696)	*p*-value
Sex (male), n (%)	1456 (52.8%)	40 (64.5%)	1416 (52.5%)	0.061
Age (years)	48.7 ± 18.1	62.2 ± 15.40	48.4 ± 18.03	< 0.001
Height (cm)	166.2 ± 9.30	165.7 ± 8.73	166.2 ± 9.30	0.707
Weight (kg)	67.8 ± 14.84	67.7 ± 14.77	72.0 ± 17.57	0.065
Co-morbidities (applicable), n (%)	1319 (47.8%)	47 (75.8%)	1272 (47.2%)	< 0.001
Duration of symptom onset (days)	4.8 ± 3.24	3.6 ± 2.71	4.7 ± 3.24	< 0.001
Total period of hospitalization (days)	12.1 ± 4.64	9.1 ± 4.32	12.2 ± 4.62	< 0.001
Vital signs				
Pulse rate (bpm)	89.1 ± 13.56	93.1 ± 13.66	89.0 ± 13.55	0.023
Saturation of percutaneous oxygen (%)	96.9 ± 1.35	96.2 ± 2.40	97.0 ± 1.32	0.017
Systolic blood pressure (mmHg)	129.1 ± 19.16	136.8 ± 17.46	129.0 ± 19.17	< 0.001
Diastolic blood pressure (mmHg)	83.9 ± 11.38	85.1 ± 9.69	83.9 ± 11.42	0.359
Laboratory findings				
White blood cell counts (× 10^3^/μL)	4.466 ± 1.5975	4.929 ± 2.0295	4.455 ± 1.5850	0.074
C-reactive protein (mg/dL)	2.73 ± 3.508	3.95 ± 4.465	2.70 ± 3.479	0.023
Blood urea nitrogen (mg/dL)	12.0 ± 4.33	14.2 ± 3.98	11.9 ± 4.33	< 0.001
Creatinine (mg/dL)	0.83 ± 0.442	0.90 ± 0.216	0.83 ± 0.446	0.015
Total bilirubin (mg/dL)	0.52 ± 1.366	0.55 ± 0.227	0.53 ± 1.382	0.484
Procalcitonin (μg/L)	0.062 ± 0.5053	0.158 ± 0.4613	0.060 ± 0.5061	0.104

The comparison of bio-signals between the two groups three and four days before discharge or exacerbation is shown in Table 2. The maximum BT three and four days before exacerbation was higher in the exacerbation group (*P* < 0.001). The minimum SpO_2_ three and four days before exacerbation was lower in the exacerbation group (*P* < 0.001). The vital bad index was 1.4 in the exacerbation group and 0.9 in the mild group (*P* < 0.001).

**Table 2:** Comparison of derived variables between transfer- and non-transfer group

Variables	Total (*n* = 2758)	Exacerbation group (*n* = 62)	Mild group (*n* = 2696)	*p*-value
Maximum BT 3 days ago (℃)	37.0	38.1	37.0	< 0.001
Maximum PR 3 days ago (mmHg)	82.9	90.9	82.9	< 0.001
Minimum SpO_2_ 3 days ago (%)	97.0	93.8	97.0	< 0.001
SBP 3 days ago (mmHg)	115.5	125.6	115.5	< 0.001
DBP 3 days ago (mmHg)	76.2	75.8	76.2	0.712
Maximum BT 4 days ago (℃)	37.0	38.1	37.1	< 0.001
Maximum PR 4 days ago (bpm)	82.9	90.6	82.9	< 0.001
Minimum SpO_2_ 4 days ago (%)	96.9	94.5	96.9	< 0.001
SBP 4 days ago (mmHg)	115.7	125.6	115.8	< 0.001
DBP 4 days ago (mmHg)	76.4	75.9	76.4	0.652
Vital bad index^a^	0.9	1.4	0.9	< 0.001

*BT* Body temperature, *PR* Pulse rate, *SpO*_*2*_, Saturation of percutaneous oxygen, *SBP* Systolic blood pressure, *DBP* Diastolic blood pressure

^a^Vital bad index: sum of bad BT score, bad PR score, and bad SpO_2_ score; Bad BT score is defined as 1 if the body temperature exceeds 38.5° three days before transfer; Bad PR score, defined as 1 if the heart rate was less than 60 bpm or greater than 110 bpm three days before transfer; Bad SpO_2_ score, defined as 1 if SpO_2_ was less than 93% three days before transfer

### Developing and evaluating models

**Fig. 2:**
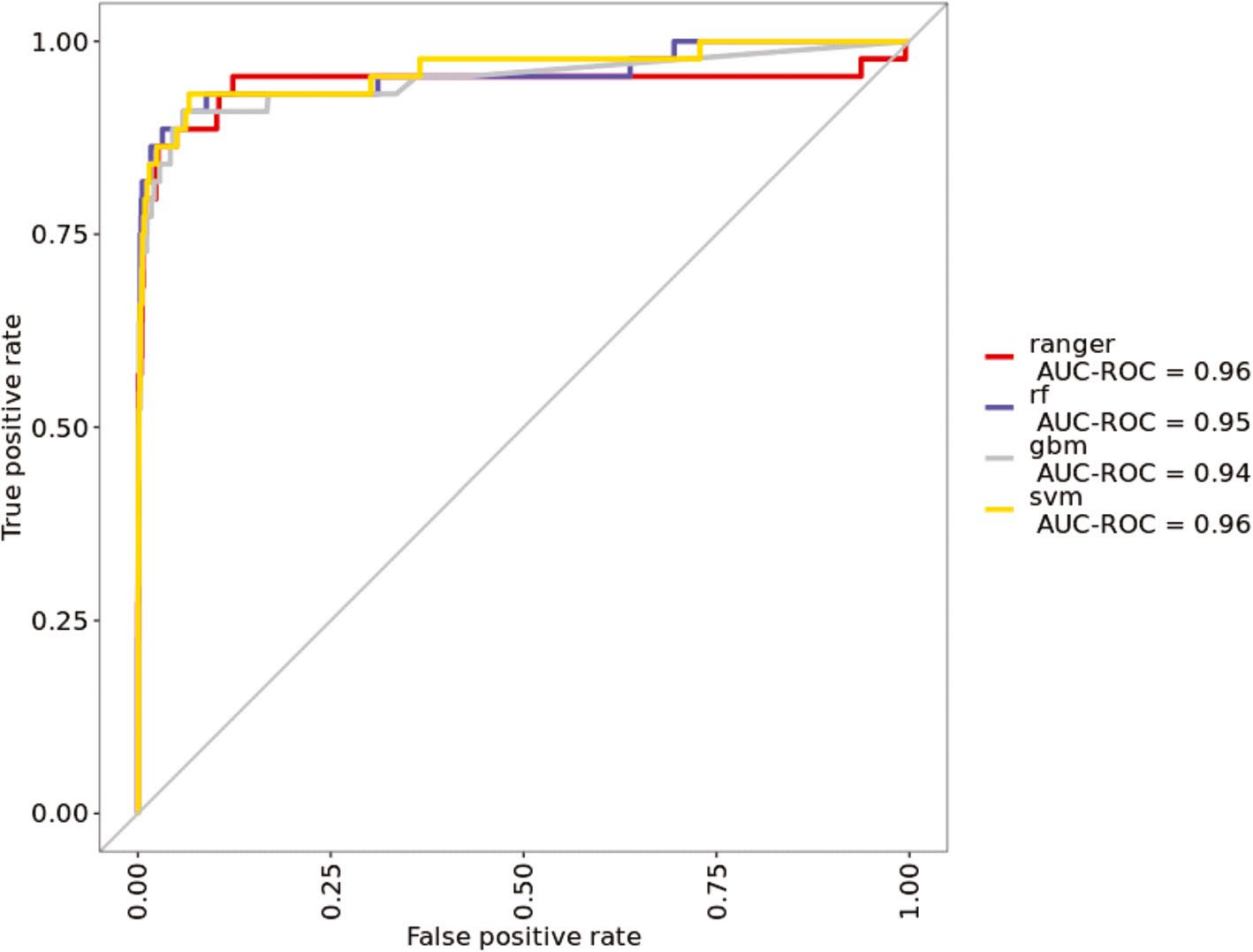
Area under the receiver operating characteristics (AUROC) of four different prediction models

In this study, four ML algorithms, RF, RF (ranger), GBM, and SVM, were trained to develop a model to predict the exacerbation of COVID-19 patients. The performance of each developed model was evaluated using accuracy, kappa, sensitivity, specificity, precision, detection rate, balanced accuracy, and AUROC as the performance metrics (Table 3, Fig. 2). According to Table 3, RF (ranger), RF, and SVM had an equal accuracy, at 0.9883. SVM had the best Kappa, at 0.703, followed by RF (0.6614). SVM had the highest sensitivity, at 0.61111. Regarding precision, RF (ranger) had 1.00000, followed by GBM and SVM at 0.84615. The detection rate was highest for SVM, at 0.01436, followed by RF (0.01175). SVM had the best-balanced accuracy, at 0.80422. Run time was the shortest for SVM, at 4.13 s, followed by GBM (7.53 s). SVM showed the best results in six of the eight evaluation indicators. Although the AUROC of the RF ranger and SVM were the same (at 0.96), the SVM algorithm performed better.

**Table 3:** Final performance of machine learning models in prediction of transfer to tertiary medical center

Performance	Accuracy (95% CI)	Kappa	Sensitivity	Specificity	Precision	Detection Rate	Balanced Accuracy	Run Time (seconds)
Random Forest (ranger)	0.9883^a^ (0.9778, 0.9946)	0.661	0.500	1.000^a^	1.000^a^	0.012	0.750	17.50
Random Forest (rf)	0.9883^a^ (0.9778, 0.9946)	0.661	0.500	1.000^a^	0.857	0.012	0.750	154.67
GBM	0.9869 (0.9761, 0.9937)	0.610	0.444	1.000^a^	0.846	0.010	0.722	7.53
SVM	0.9883^a^ (0.9778, 0.9946)	0.704^a^	0.611^a^	0.997	0.846	0.014^a^	0.804^a^	4.13^a^

*GBM* Gradient boosting machine, *SVM* Support vector machine

^a^Best result of each model

**Fig. 3:**
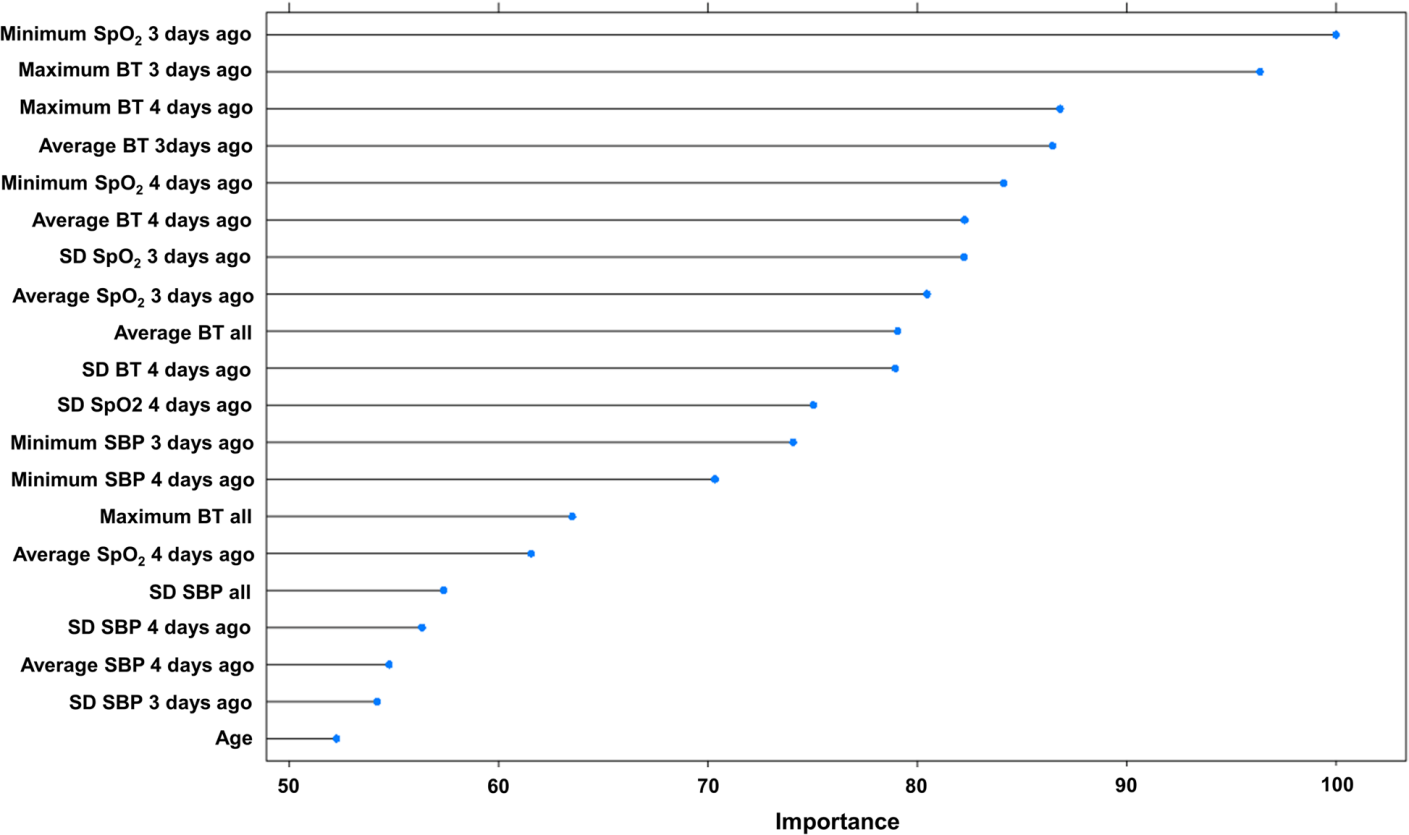
Top 20 variable importance of support vector machine

Among a total of 100 variables, 86 were used for all machine learning methods upon preprocessing. The top 20 variables important in SVM are shown in Fig. 3. BT, SpO_2_, and SBP were included in the top 20 important variables. The minimum SpO_2_ 3 days before had the highest feature importance, followed by the maximum BT 3 days before, the maximum BT 4 days before, and the average BT 3 days before.

## Discussion

In this study, we developed an algorithm that can predict the likelihood of a mild-to-severe COVID-19 patient being exacerbated to a tertiary medical institution equipped with tracheal intubation, ventilator, and negative pressure isolation room three days in advance. We analyzed continuous data from individual patients to predict the exacerbation of symptoms. With this study, we can help medical staff in deciding whether to exacerbation a patient to a tertiary hospital. In addition, the timing was predicted in advance. Recently, many studies have developed predictive models for the worsening of COVID-19 patients [15–18]. The characteristic of our study is that it developed a model for predicting the worsening of COVID-19 patients as well as simultaneously providing a warning in advance to the medical staff of whether the patient may need to be exacerbated to a tertiary hospital for advanced treatment. Although our model used easy-to-measure bio-signals, the AUROC was 0.96, which is a fairly high accuracy. Regarding variable importance, BT and SpO_2_, which are generally easy to measure, showed high importance. Currently, if the number of COVID-19 patients rapidly increases, medical institutions are unable to manage all patients with current resources. In Korea, asymptomatic or mildly symptomatic patients self-check their symptoms at home and are hospitalized only when their subjective symptoms worsen. However, for non-medical patients determining the need for inpatient treatment is difficult, therefore, the application of our proposed model can ensure that patients with mild symptoms can receive timely treatment. Medical resources, such as negative pressure isolation inpatient wards, mechanical ventilators, and intensive care units, cannot cope with the rapidly increasing number of patients. Therefore, if the deterioration in the condition of COVID-19 patients can be accurately predicted at an early stage, limited medical resources can be efficiently utilized.

According to an April 2021 review article on predicting the mortality and severity of COVID-19 using ML, the most used models were logistic regression, followed by extreme gradient boosting and SVM [19]. However, many methods are being attempted but the accuracy has remained unchanged for each method. According to literature, the SVM model was developed using laboratory findings as variables to predict severity; however, in such a pandemic situation, predicting the prognosis through the results of blood tests is cumbersome and relatively difficult to apply to actual clinical practice because there is a limit in terms of time and cost. Therefore, our study will be more useful in the current pandemic situation as the vital signs and SpO_2_, which are relatively easy to obtain, are the main variables.

Although this study shows the possibility of developing a model that can be easily applied to clinical practice, it has several limitations, the first of which is its implementation in a single institution. Since COVID-19 has not yet ended, we intend to undertake further research related to COVID-19, in which multi-center COVID-19 patient data will be collected and external validation will be conducted. Second, the variable SpO_2_ alone was considered in model development without information on oxygen supply. The ratio of SpO_2_ and supplied O_2_ flow more accurately reflects the medical condition of the patient. Thus, this is planned as a further study. Third, data from asymptomatic COVID-19 patients who were not hospitalized were not collected; this is also planned as a further study in the future. If data on asymptomatic patients are collected, the performance of the exacerbation model can be improved.

## Conclusions

In conclusion, predicting deterioration in advance so that appropriate treatment can be achieved is an important diagnosis of COVID-19 for improving the survival rate of patients. Our model predicts with high accuracy 3–4 days before the condition of the patient with COVID-19 worsens. Therefore, this algorithm can facilitate adequate oxygen therapy and mechanical ventilator preparation, thereby improving patient prognosis, increasing the efficiency of the medical system, and mitigating the damage caused by the global pandemic.

## Data Availability

The datasets generated and/or analysed during the current study are available in the GitHub repository. [https://github.com/HanEol-Lee77/Prediction-Model-of-Covid-19-Exacerbation].

## Abbreviations

COVID-19: 2019 Coronavirus
ICU: Intensive care units
ML: Machine learning
EMR: Electronic medical records
SBP: Systolic blood pressure
PR: Pulse rate
BT: Body temperature
SpO2: Percutaneous oxygen saturation
BUN: Blood urea nitrogen
RF: Random forest
GBM: Gradient boosting machine
SVM: Support vector machine
AUROC: Area under the receiver operating characteristics
